# Associations between community violence and pediatric sleep health: A systematic review

**DOI:** 10.1016/j.sleh.2025.10.009

**Published:** 2025-11-25

**Authors:** Hannah R. Scheibner, Eileen M. Condon, Kelley A. LaFleur, Nancy S. Redeker

**Affiliations:** School of Nursing, University of Connecticut, Storrs, Connecticut, USA

**Keywords:** Community violence, Neighborhood, Sleep health, Pediatrics, Social determinants of health, Systematic review

## Abstract

Inadequate sleep during childhood can have a lasting negative impact on lifelong health. Children living in disadvantaged neighborhoods are more likely to be exposed to community violence, which may disrupt sleep health. The purpose of this systematic review was to examine the associations between community violence and pediatric sleep health. We searched 4 electronic databases (CINAHL, Scopus, PubMed, and Embase) to identify articles that examined the associations between community violence and pediatric sleep health. We screened 2271 articles and included 29 eligible studies. Studies focused on sleep quality (n = 4), daytime sleepiness (n = 8), sleep timing (n = 4), sleep efficiency (n = 7), duration (n = 13), and sleep disturbance (n = 19). Most studies included adolescents (n = 18); others included infants (n = 1), those in early childhood (n = 2), and a wide range of ages (n = 7). Six studies were of fair quality, while 5 were of poor quality, often due to the use of measures that were not psychometrically sound and incomplete sample descriptions. Community violence exposure was consistently negatively associated with pediatric sleep health. Consistent definitions and measures of sleep and community violence are needed to promote rigor and comparisons across studies. Future studies should include infants and children under the age of 6 years and address potential risk and protective factors for sleep health. Research is needed to inform policy changes and multilevel community-based interventions to help buffer the harmful effects of community violence and promote pediatric sleep health.

## Introduction

Healthy sleep is essential for children’s health and development, laying the foundation for lifelong well-being and good sleep habits.^1^ Inadequate sleep is characterized by short, fragmented, poorly timed sleep and sleep disorders. On the other hand, sleep health includes positive sleep behaviors, satisfaction, daytime alertness, timing, efficiency, and duration (Table 1).^2,3^ Lack of adequate sleep during childhood can lead to negative health effects throughout life, such as increased cardiometabolic risk and impaired memory, attention, academic performance, and emotional and behavioral functioning.^1,4^ Several social stressors are known to affect children’s sleep health, including parent mental health and family routines.^5,6^ However, less is understood about how environmental stressors impact child sleep. Gaining a better understanding of these relationships will help develop interventions to promote sleep health among children exposed to environmental stressors.

One major environmental stressor that may influence children’s sleep is exposure to community violence. Community violence occurs outside the home among unrelated individuals who may or may not know each other (Centers for Disease Control and Prevention^7^). Community violence may directly disrupt sleep due to noise and disturbances associated with shouting, gunfire, sirens, an increased police presence, and other factors.^8,9^ Community violence exposure may induce stress, fear, anxiety, and hypervigilance, which may lead to later bedtimes, difficulty falling asleep, and shorter sleep duration. While these responses constitute evolutionary survival mechanisms to support accurate assessments of safety while asleep,^10^ they may contribute to worse sleep quality and sleep disturbances. Other social factors that may co-occur with or arise from exposure to community violence, such as crowded living conditions, poverty, trauma, and caregiver mental health, may also account for the associations between community violence and inadequate sleep.^11,6,12^ Understanding the relationship between community violence and pediatric sleep health is therefore essential for identifying at-risk groups, creating interventions to support healthy sleep, and ultimately promoting overall health and well-being.

Prior reviews have demonstrated associations between neighborhood environments and child sleep,^13^ and have identified community violence as a potential pathway linking environments to sleep health and disorders among children and adults.^8^ The purpose of the present review is to build on these findings by explicitly focusing on the relationship between community violence exposure and child sleep health. To our knowledge, this is the first systematic review on this topic. Framed by the Social-Ecological Model of Sleep Health (SEMOSH), the goal of this systematic review is to inform future research and the development of multilevel community-based interventions to promote child sleep health.^14,9^

We addressed the following aims:

1. Examine the association between exposure to community violence and dimensions of pediatric sleep health and sleep disturbance.
2. Assess the quality of included studies, including samples, measures, and methods.
3. Identify implications for future research and multilevel interventions.

## Methods

### Data sources and search strategy

We systematically reviewed the literature and selected articles in accordance with the Preferred Reporting Items for Systematic Reviews and Meta-Analyses (PRISMA) guidelines. We collaborated with a research librarian to develop the search strategy and methods. We defined community violence as “violence… between unrelated individuals, who may or may not know one another, generally outside of the home”.^7^ We used the B-SATED definition of pediatric sleep health to guide our selection of terms related to pediatric sleep health, including sleep, sleep latency, nightmares, and sleep quality.^3^ Table 1 outlines each dimension of B-SATED.

### Inclusion and exclusion criteria

We included empirical studies that examined the relationships between community violence and sleep health in pediatric populations. We included studies that measured any dimension of sleep using objective or self-report measures to capture a complete representation of the literature. We selected studies published in English that included participants aged 0–17 years, measured relationships between community violence and sleep health, and had publication dates in any year. We excluded studies of interpersonal violence, bullying, domestic violence, politically motivated rape, wartime, forced displacement into refugee camps, and natural disasters.

### Article screening and citation management

After removing duplicates, all articles were screened at the title and abstract and full-text levels by at least 2 authors. All 3 reviewers discussed any conflicts concerning inclusion or exclusion decisions until we reached a consensus. We used the Rayyan.ai systematic review platform for online citation screening.^15^ We extracted data on study design, methods, measures, and demographic characteristics into a table for all included studies.

### Study quality assessment

We used the Joanna Briggs Institute (JBI) Critical Appraisal Checklist to assess the sample description, measures, confounding factors, and statistical analysis methods. We rated studies as good quality if they met all JBI criteria, fair quality if they did not meet 1–2 criteria, and poor quality if they did not meet 3 or more criteria.^16^

## Results

### Search results

**Fig. 1.**
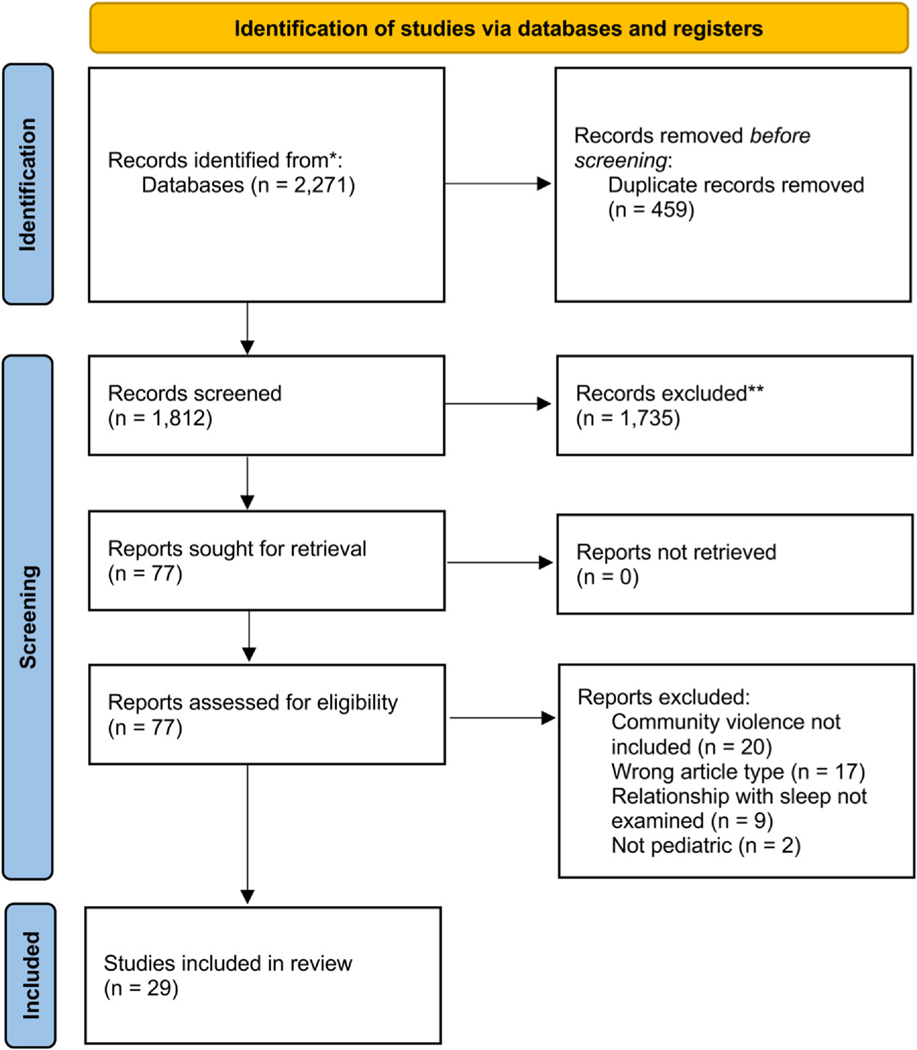
PRISMA flowchart for study selection. PRISMA, preferred reporting items for systematic reviews and meta-analyses Source: Page MJ, et al. BMJ 2021;372:n71. doi: 10.1136/bmj.n71. This work is licensed under CC BY 4.0. To view a copy of this license, visit https://creativecommons.org/licenses/by/4.0/.

The electronic database search yielded 2271 unique citations. Twenty-nine studies were included (Fig. 1). After removing 459 duplicates, reviewers screened 1812 unique citations at the title and abstract levels. We assessed 77 articles at the full-text level. We excluded the remaining 48 because they did not address community violence (n = 20), were of the incorrect article type (e.g., abstract only, qualitative) (n = 17), did examine the relationship with sleep (n = 9), or did not involve pediatric populations (n = 2).

Table 2 describes the included studies. The studies were conducted in the United States (n = 24), Canada (n = 3), and Norway (n = 1). One did not mention a location. Most studies included adolescents (n = 19), while others included children in infancy (n = 1), early childhood (n = 2), and across a wide range of ages (n = 7). Studies included a total of 138,850 participants.

### Study quality assessment

Eighteen studies were of good quality and met all JBI criteria (see Table 3). Six studies were of fair quality, and 5 were of poor quality. Poorer study quality was often due to the use of nonstandard and invalid measures of community violence or sleep health, or inadequate sample description (location, race, and ethnicity).

### Community violence measures

Measures of community violence varied across studies (see Table 2). Two out of 29 studies employed objective measures, such as geocoded crime data and police crime reports,^9,17^ while 28 included self-report measures. Single items or subscales from validated instruments were often used. Fourteen different questionnaires were used to measure community violence. Eleven included self-report questionnaires, while 3 included parent-report of community violence. Indicators of community violence included reports of robberies, physical assaults, or participant-witnessed neighborhood violence.

### Sleep variables and measures

The types and quality of sleep health measures varied widely across studies (see Table 2). Among the 29 studies, 6 employed both self-report and objective sleep measures. These included wrist actigraphy^18,9,19–21^ and clinician assessments.^22^ One study used only objective sleep measurement (wrist actigraphy).^23^ Twenty-two studies used self-report sleep measures. Selected items or subscales from validated instruments were often used rather than complete instruments. Fifteen different questionnaires were used to assess sleep. Eleven studies included self-report questions, while 4 included parent-report questions regarding sleep.

### Relationship between community violence and sleep

We organized the findings based on sleep dimensions. We used the B-SATED definition of pediatric sleep health whenever possible.^3^ We also added a sleep disturbance category for findings that did not fit into the B-SATED model, such as general sleep difficulties or problems, insomnia symptoms, and nightmares. Overall, the studies included in this review looked at sleep quality (n = 4), daytime sleepiness (n = 8), sleep timing (n = 4), sleep efficiency (n = 7), sleep duration (n = 13), and sleep disturbance (n = 18). No studies examined the link between community violence and sleep behaviors.

### Sleep quality

Four studies (13.8%; N = 1328) included measures of sleep quality and were conducted among adolescents, yielding consistent findings.^18,24,23,25^ Exposure to community violence was associated with poorer sleep quality among racial and ethnic minority adolescents (b = 0.64, p < .05, N = 151).^25^ Adolescents with poor sleep quality were more than 5 times as likely to have experienced community violence compared with those who were not exposed.^24^

### Daytime sleepiness

Eight studies included measures of daytime sleepiness (27.6%; N = 2333) using descriptors such as sleepiness, overtiredness, naps, and sleep/wake problems.^18,26,27,25,28,29,12,21^ In 7 of these studies, exposure to community violence was associated with daytime sleepiness or increased napping among adolescents^18,26,27,25,29,21^ and children.^12^ A statistically significant association was found between community violence exposure and sleepiness in schools (r = 0.62, p < .001, N = 498).^26^ Other studies, however, included measures of daytime sleepiness in various environments. In contrast, there was no statistically significant association between community violence and sleepiness among Black adolescents (r = 0.04, N = 101).^28^ The association between community violence and sleepiness (N = 323) was buffered by socioeconomic indicators, such as a high income-to-needs ratio (p < .001) and perceived financial stability (p < .001).^21^ Associations between community violence and sleepiness were mainly consistent, and protective factors may buffer this relationship.

### Sleep timing

Four studies included measures of sleep timing (13.8%; N = 544), with 2 studies finding a significant association between community violence and later sleep timing. Exposure to violent crime and feeling unsafe in neighborhoods were associated with later bedtimes among children aged 11–13 (r = 0.57, p = .001, N = 54)^30^ and aged 11–18 (M = 26 minutes, p = .043, N = 82).^9^ When a violent crime occurred within 0.5 miles of a child’s home (based on geocoded data), bedtimes were 30 minutes later compared with crime occurring farther away (p = .036).^9^ Mean and median bed and wake times for adolescents aged 11–14 exposed to community violence were reported (N = 362), but the study did not examine the association.^31^ Associations between witnessing a homicide and sleep timing were inconsistent. There were significantly later bedtimes (1.81 h, robust SE = 0.198, p < .001) among those who witnessed a homicide in one study.^9^ Yet no association was found in another.^20^

### Sleep efficiency

Seven studies included measures of sleep efficiency and latency (24.1%; N = 3610) across all age groups; however, the results were inconsistent. Exposure to community violence was associated with reduced sleep efficiency among adolescents, while controlling for anxiety and demographic variables (B = −1.68, SE =.60, p < .01, N = 252).^18^ Race (African American, European American) moderated the relationship between community violence concerns and sleep efficiency, such that African American adolescents with higher violence concerns experienced lower sleep efficiency.^23^ Income moderated the relationship between neighborhood safety and sleep efficiency (β = −.15, p < .05, N = 323). Perceived lower neighborhood safety was associated with lower sleep efficiency among adolescents from lower-income households.^21^ Police community crime reports and mothers’ perceived lack of safety were correlated with reports of less consolidated sleep among infants (r = −0.085, p < .01, N = 2445).^17^ In contrast, 3 studies found no associations between community violence exposure and parent-reported sleep latency among infants and children aged 11–18.^9,31,17^

### Nighttime awakenings

Five studies included measures of nighttime awakenings (17.2%; N = 3309), but the findings were inconsistent. Waking after sleep onset (WASO) was higher among children exposed to community violence or those who witnessed homicides,^19–21^ with 73.6% of children ages 11–14 (N = 362) who experienced community violence reporting night waking.^31^ Neighborhood safety was associated with lower WASO (b = −8.60, SE = 3.34, p = .01, N = 133) only among American Indian or Alaska Native adolescents who reported thoughts of historical trauma, indicating a significant moderation effect.^19^ In contrast, there were no associations between violence and nighttime awakenings among infants (r = 0.034, N = 2445)^17^ and children ages 4–17 (β = 0.01, SE = 0.07, CI (−0.12, 0.11), N = 10,802).^32^

### Sleep duration

Thirteen studies (44.8%; N = 67,274) included measures of sleep duration. Ten revealed associations between exposure to community violence and insufficient sleep duration.^18,33,9,34,17,27,35,23,20,21^ Six of the 13 studies included wrist actigraphy to measure sleep duration.^18,9,19,23,20,21^

Witnessing homicide was associated with shorter self-reported sleep duration among children aged 8–16 (GMR = 1.49, CI (.96, 2.30), p < .10, N = 46),^20^ and with shorter actigraphy-measured sleep duration among children aged 11–18 (1.14 h, p = .000, N = 82).^9^ Among infants, sleep duration decreased by 10 minutes with a one standard deviation increase in police crime reports and by 80.34 minutes with a one standard deviation increase in mothers’ perceived lack of safety.^17^ However, children aged 6–18 years exposed to community violence had longer sleep (caregiver report) (d = 0.37, N = 276).^12^

Differences in sleep duration after exposure to community violence varied by sex (N = 262),^18^ race (N = 219),^23^ risk and protective factors (e.g., mental health, substance use, physical activity, and connectedness) (N = 17,033),^33^ and financial status and stability (N = 323).^21^ Males, African Americans, and individuals with lower incomes had shorter sleep duration than their female, White, and higher-income peers.

One study included measures of perceived safety in school and neighborhood environments among adolescents (N = 7958).^35^ Adolescents who felt unsafe at school and in their neighborhoods had 129% greater odds of insufficient sleep duration than peers who felt safe in both locations.^35^ Compared with adolescents who felt safe in both locations, those who felt unsafe only in their neighborhood had 71% greater odds of insufficient sleep duration, while those who felt unsafe only in school had 39% greater odds.^35^

### Sleep disturbance

Eighteen studies included measures of general sleep disturbance that did not fit into the B-SATED model, such as general sleep difficulties or problems, insomnia symptoms, and nightmares (62.1%; N = 75,040). Seventeen of these studies identified an association between community violence and sleep disturbance.

### Sleep problems

In 7 studies (N = 46–20,716), witnessing or being a victim of community violence was associated with unspecified sleep problems among children aged 6–18 years.^36,22,31,26,37,20,29^ Four longitudinal studies detected lasting effects of community violence exposure on pediatric sleep problems. Exposure to community violence predicted future sleep problems at 3 timepoints among adolescents (ages 13, 16, and 17) (Time 1: β = 0.74, Time 2: β = 0.74, Time 3: β = 0.55, all p < .05, N = 84)^37^; in a latter year among children and adolescents (ages 9–18) (b=0.041, SE=0.017, p < .05, N = 20,716)^38^; and for 3 months following the event among children and adolescents (aged 8–16) (MD = 4.45, SE = 1.76, p < .05, N = 46).^20^ Another study identified an indirect pathway where violence exposure led to difficulty talking about violence (β = 0.16, p < .01), which led to intrusive thoughts (β =.20, p < .1), and subsequent sleep problems (β =.11, p < .05; N = 327).^31^ Among adolescents aged 13–18 (N = 4043), 33.6% reported exposure to community violence, which was associated with sleep problems.^22^ However, 10.5% of children aged 7–12 were exposed to community violence, but the association with sleep problems was not statistically significant.^22^

In 2 studies employing self-report measures, sleep disturbances were not associated with neighborhood violence (r = 0.07, N = 101)^28^ or neighborhood safety (β = −.06, p = .107, N = 7932) among children and adolescents.^39^

### Insomnia symptoms

Associations between community violence and insomnia symptoms were examined in 3 studies (10.3%; N = 12,403), measured by self-reported difficulty falling asleep on most or all nights. However, the findings were inconsistent.^40,28,29^ Among adolescents, community violence exposure was associated with insomnia symptoms (p < .05, N = 263)^29^ and across a 22-year follow-up period to mid-adulthood (p < .05, N = 12,039).^40^ However, the odds for the association between violence and insomnia (OR 1.53, CI [1.26, 1.87]) were no longer statistically significant when controlling for behavioral covariates (e.g., depression, smoking) (OR 1.0, CI [0.75, 1.33]).^40^ In contrast, neighborhood violence was not associated with insomnia symptoms among adolescents (r = 0.07, N =101).^28^

### Nightmares

Investigators examined nightmares in 3 studies (10.3%; N = 21,255).^29,38,12^ Among children and adolescents, exposure to community violence was associated with nightmares to varying degrees (r =.131, p < .010, N = 263),^29^ compared with those not exposed to community violence (t = −1.72, p = .08, d = 0.36, N = 247).^12^ Another study asked one question about nightmares within a sleep troubles questionnaire, but reported no results or associations for this item.^38^ These findings demonstrate an association between community violence and nightmares among children and adolescents.

## Discussion

We found that community violence is associated with poor pediatric sleep health, including poorer sleep quality, increased daytime sleepiness, later bedtimes, lower sleep efficiency, shorter duration, and greater disturbance. The results were more consistent for some sleep dimensions, such as quality, duration, and disturbance, while findings for timing, efficiency, and daytime sleepiness were less consistent with null or conflicting findings. Sleep duration and disturbance were the most studied sleep dimensions, whereas timing, efficiency, daytime sleepiness, and quality were less commonly studied, which may contribute to the inconsistent findings for these dimensions. Associations were strongest for sleep duration, with exposure to community violence linked to shorter sleep duration.

The relationship between community violence and sleep health has been primarily studied in adolescents, with few studies examining this association among infants and children under 6 years of age. Thus, most findings are limited to adolescents. In studies that included children of various ages, developmental differences in sleep characteristics and perceptions of community violence were often unexamined, reducing rigor and possibly confounding the results.

Most (62%) of the studies reviewed were of good methodological quality and included diverse samples in terms of age and race or ethnicity, which enhanced the generalizability of findings. Some studies provided specific details about the geographical regions examined (i.e., states, cities, and urban areas), while others were less explicit. A frequent limitation of the studies included in this review was the lack of valid, reliable, and consistent measures of community violence and sleep. Most studies included self-report measures of sleep and community violence, many of which have not been validated for use among children and adolescents. Single-item measures, coupled with inconsistent use of self- versus parent-report, limited the rigor of some studies. In addition, wide variation in the definitions and measures used reduced our ability to synthesize the results. Thus, despite the methodological strengths of the studies reviewed, inconsistencies in reporting study sample, setting, and measurement validity are important limitations of existing literature.

Self-reports of both community violence and sleep may be influenced by an individual’s negative affectivity (e.g., anxiety, stress), which may increase one’s perception of community violence or poor sleep, or function as a mediator by linking violence exposure to poorer sleep.^31,41^ Children’s exposure to caregiver stress may increase their perception of community violence, while children who are protected from violence by their caregivers may be less aware and less accurate reporters of violence in their communities. Use of objective measures of community violence (geographic information systems, crime or violence data, and geocoded self-report data) may overcome some limitations of self-reports. However, area-level metrics may not reflect participants’ direct experiences. Similarly, for sleep measurement, use of wrist actigraphy with sleep diaries can provide more objective measurements of sleep timing, efficiency, and duration. In contrast, self-report measures of sleep offer essential information on sleep behaviors, perceptions, and quality. Use of a combination of objective and self- and parent-report measures will advance understanding of how exposures, perceptions, and experiences intersect to form relationships between community violence and pediatric sleep health.

Beyond measurement, conceptual gaps in the literature further limit understanding of this phenomenon. Our ability to compare findings across studies was limited by lack of consistent definitions for sleep health dimensions, particularly sleep quality and disturbance, and limited use of theoretical frameworks for sleep and community violence. Notably, no studies investigated possible relationships between community violence and sleep behaviors such as routines, bedtime interactions, or electronic use, despite these being modifiable behaviors that can be targeted in interventions.^3^ Sleep behaviors, such as bedtime routines, are essential for young children and may buffer them against the adverse effects of community violence.^5^ Among older children, adolescents living in neighborhoods with high crime reported more screentime,^42^ possibly as a coping strategy for nighttime anxiety and fear. Similarly, community violence may undermine families’ ability to engage in healthy sleep behaviors—including parents’ bedtime routines, perceptions of safety, and bedtime interactions—through anxiety, fear, stress, or hypervigilance.^43^ These unexplored modifiable factors should be considered as potential confounders or mechanisms linking community violence to pediatric sleep.

Most (72.4%) of the reviewed studies were cross-sectional in design, which restricted understanding of the temporal or causal links between violence and sleep. We found inconsistent results related to community violence exposure severity, geography, and factors such as demographics, school environments, attitudes, behaviors, and risk and protective factors that may be linked to sleep health outcomes in children.^40,14,26,19,23,21^ Heterogeneity of samples across studies and unmeasured potential confounders or mediators, such as mental health and family factors, may have contributed to these inconsistencies and highlight study design limitations.

### Future directions for research

Our systematic review highlights several directions for advancing research to further understand the relationship between community violence and pediatric sleep health. Establishing a consistent theoretical framework and conceptual definitions—such as adopting the B-SATED definition of pediatric sleep health—will enhance conceptual clarity and comparability of studies. Utilizing a combination of valid and reliable objective and self-report measures can elucidate pathways linking community violence to pediatric sleep health, while including measurements of negative affect, mental health, and family factors may clarify potential mechanisms.^14^ Together, these approaches will strengthen study quality and expand our evidence base to inform interventions.

Conducting age-specific research among infants and younger children is crucial, as this population requires the longest sleep duration, learns sleep habits and behaviors from their parents or caregivers, and undergoes formative growth and development that establishes the foundation for lifelong health.^1,44^ Because self-report is not feasible among infants and young children, reliable objective measures of sleep and community violence are important. Among studies of children across a wide range of ages, developmental stage should be considered in the design, analysis, and interpretation of results.

Future studies should adopt longitudinal designs with larger sample sizes and continue to include diverse populations to strengthen power and study design. Longitudinal studies beginning in infancy or early childhood may further uncover relationships between community violence and sleep health. Recruiting larger and more diverse samples may clarify potential moderators of the relationships between sleep and violence, such as sex, race, financial status, and historical trauma.^18,39,19,23,45,21^

### Implications

Findings from this review highlight opportunities for multilevel interventions to promote healthy sleep among children exposed to community violence. While some programs address stress-related sleep problems, few focus on community violence or are tailored to racially and ethnically diverse pediatric populations.^46^ At the policy level, reforms such as community reinvestment, delegating specific police duties to social workers, and gun safety legislation address root causes.^47,48^ Schools and community organizations can strengthen these efforts through sleep hygiene education, mentorship, and support services.^49,46,26,48,50^ At the individual level, age-appropriate strategies such as mindfulness, relaxation, and safety-promoting practices can help buffer children against the effects of community violence, supporting resilience and sleep health equity.^14^

### Strengths and limitations of the review

Our review identified only English-language studies from the United States, Canada, and Norway and did not include gray literature. Studies published in languages other than English or conducted in countries outside of North America and Europe may exist but were not captured in this review. This highlights the need for more international work and descriptions of geographic location and urbanicity.

The strengths of this study include a rigorous approach, a clear theoretical framework (SEMOSH)^14^ that guided all elements of the review, and well-accepted definitions of pediatric sleep health^3^ and community violence,^7^ which informed both the search strategy and the interpretation of results.

## Conclusions

Children’s exposure to community violence is associated with suboptimal pediatric sleep health. To advance understanding, future research should focus on understudied sleep health dimensions (timing, efficiency, sleepiness, quality, and behaviors), children under age 6, the influence of covariates (demographic, structural, and behavioral factors), and longitudinal patterns. The creation and testing of interventions at individual, community, and societal levels are critical to promoting pediatric sleep health equity.

## Supplementary Material

Supplementary material

## Figures and Tables

**Table 1:** Summary of B-SATED definition of pediatric sleep health

Sleep health dimension	Definition

Behaviors	Actions or activities that may promote or inhibit sleep health
Satisfaction/Quality	Subjective assessment of “good” or “poor” sleep
Alertness/Sleepiness	Ability to maintain attentive wakefulness
Timing	The placement of sleep within the 24-hour day
Efficiency	Ease in which one can fall or return back to sleep
Duration	Amount of sleep that occurs within the 24-hour day

**Table 2:** Summaries of included studies in the review

Authors, year	Sample size (N)	Location	Age, M, (SD), sex	Race/ethnicity	Sleep dimension	Sleep measures	Community violence measures	Key findings

Bagley et al., 2016	252	Southeastern United States	M = 15.79 y (0.81), 53% female	66% European American, 34% African American	Satisfaction/Quality, Daytime Sleepiness, Duration, and Disturbance	Actigraphy, subscales of School Sleep Habits Survey (sleep/wake problems, sleepiness)	Self-report crime safety subscale of Neighborhood Walkability Scale	More sleep/wake problems, long wake episodes, and lower sleep efficiency were predicted by community violence
Bailey et al., 2005	268	Detroit, MI area, U.S.	6–7 y, M = 6.9 y, 51% female	100% African American	Disturbance	Self-report, “How often do you have difficulty sleeping?”	Self-report Things I Have Seen and Heard Scale	Risk of sleep problems increased 94% with experience of community violence. Difficulty sleeping is associated with witnessing and victimization
Baiden et al., 2024	17,033	United States	14–18 y, 48.3% female	Non-Hispanic White 54.6%, Non-Hispanic Black 13.9%, Hispanic 19.1%, Asian 5%, NA/AI 1.4%, and Other 6%	Duration	Self-report, “On an average school night, how many hours of sleep do you get?”	Self-report, “Have you ever seen someone get physically attacked, beaten, stabbed, or shot in your neighborhood?”	Higher odds of insufficient sleep duration when exposed to community violence when controlling for demographics, protective, and risk factors
Birkeland et al., 2022	4921	Norway	6–18 y M = 14 (2.7), 63.7% female	Not stated	Disturbance	Self-report (post-traumatic stress symptoms)	Self-report: “assault, robbery, violence or threats of violence outside the family” and/or “witnessing violence outside the family”	Sleep difficulties were experienced when community violence was experienced
Cai et al., 2024	7932	United States	M = 9.93 y (0.63), 49% female	Not stated, nationally representative	Disturbance	Sleep Disturbance Scale for Children	Crime Scale of Neighborhood Characteristics Measure	Increased neighborhood safety was not associated with decreased sleep disturbance over time
Desch et al., 2023	12,039	United States	12–18 y, 50% female	Not stated, nationally representative	Disturbance	Self-report, difficulty falling or staying asleep ≥ 3–4 times a week	Individual Adverse Childhood Experience exposure	Community violence exposure was associated with insomnia over 22-year follow-up. History of community violence exposure had 1.48 higher odds of insomnia and 1.53 higher odds adjusting for demographics. This relationship was no longer significant when also adjusting for potential mediators (health/risk behaviors)
Dowdell & Santucci, 2003	54	Southwest section of Philadelphia, PA, U.S.	11–13 y, 62% female	87% Caucasian, 7% African American, 4% Hispanic, and 2% other minorities	Timing	Self-report bedtime	Self-report rating of community violence, Youth Risk Behaviors Surveillance System Questionnaire (YRBSS)	Feeling less safe in the neighborhood is correlated with later bedtime
Hall Brown et al., 2016	4043	United States	7–18 y, 55.3% female	56.8% White	Disturbance	Clinician assessment (e.g., LCSW, MD, and PhD), reports from other providers, and parent-report: generated global ratings indicating degree of sleep problems (DSM-IV)	Self-report Trauma History Profile (THP) from the trauma history component of UCLA PTSD-RI	Exposure to community violence independently contributed to risk of sleep disturbance
Heissel et al., 2018	82	Large U.S. Midwestern city	11–18 y M = 14.9, 49% female	17% Black, 20% Hispanic White, 18% non-Hispanic White, and 27% multiethnic	Timing, Efficiency, and Duration	Actigraphy and sleep diaries	Geocoded crime data from police department	Children had later bedtimes on nights following violent crime. The distance of crime to home corresponded with later bedtimes
Jackson et al., 2024	345	Baltimore City, MD, U.S.	12–21 y M = 17.83, 47.25% female	100% Black or African American, 3.48% multiracial	Disturbance	Self-report “about how long do you sleep most nights?”, “how many nights out of 7 in a typical week do you have problems a) falling asleep and b) staying asleep.”, summed into composite measure of sleep disturbances	Self-report, frequency of viewing digital media videos of police encounters, whether they knew if fatality occurred	Viewing nonfatal police violence videos increased odds of trouble falling asleep (OR=2.42), viewing fatal videos further increased odds of trouble falling asleep (OR=4.48) and staying asleep (OR=6.83). About 30% of this association is attributable to emotional distress
Kliewer & Lepore, 2015	362	Philadelphia, PA and Richmond, VA, U.S.	11–14 y M = 12.45 (0.59), 51.1% female	51% Latino/a, 34% Black, 8% White, 5% Native American, and 6% Asian	Timing, Efficiency, Duration, and Disturbance	Self-report Sleep/Wake Behavior Problems Scale	Self-report modified version of Survey of Children’s Exposure to Community Violence	Witnessing violence contributes to poor sleep
Kliewer et al., 2019	107	Low-income urban areas of central Virginia, U.S.	M=14.29 y, 56% female	100% African American	Disturbance	Self-report Sleep/Wake Behavior Problems Scale	Self-report Survey of Children’s Exposure to Violence	Significant positive associations between community violence exposure and sleep disruption
Langevin et al., 2024	706	Quebec, Canada	14–18 y, 100% female	Canadian 88%, Indigenous/Metis 3.4%, Latinx 4.0%, Black 3.7%, Asian 2.8%, West European 8.8%, East European 2.1%, Caribbean 2.5%, North African/Middle Easterner 2.5%, and Other 0.3%	Quality	Pittsburgh Sleep Quality Index	Self-report “Outside of your family, have you seen someone being attacked or intentionally hurt with an object that could hurt them?”	Community violence exposure was correlated with every sleep quality dimension, except efficiency and higher odds of poor-quality sleep than moderate-quality (OR=1.936) and high-quality sleep (OR=5.408)
Lepore & Kliewer, 2013	498	Philadelphia, PA and Richmond, Virginia, U.S.	M = 12.8 y (0.44), 56% female	43% White, 24% Latinx, 24% African American, and 9% other	Daytime Sleepiness, Disturbance	Self-report Sleep/Wake Behavior Problems Scale	Self-report modified version of Survey of Children’s Exposure to Community Violence	Sleepiness in school may indicate exposure to community violence
Lin et al., 2022	46,209	United States	6–17 y, sex not reported	Not stated, nationally representative sample	Duration	Parent-report average weeknight sleep duration	Individual Adverse Childhood Experience exposure	Prevalence of insufficient sleep duration is higher in witnesses/victims of neighborhood violence
MacKinnon et al., 2021	2445	Calgary, CA	12 m, 100% female (mothers)	Only reported for mothers (76.8% White, 13.1% Asian, 2.3% Latinx, 1.5% African American, 1.5% Arab, 0.8% American Indian, and 3.6% mixed/other)	Efficiency	Mother reported	Police community crime reports, mother rated perceived safety of neighborhood	Less sleep consolidation was predicted by perceived maternal unsafety and neighborhood disorder
McHale et al., 2011	469	Southwestern metropolitan area, U.S.	7th graders (age not reported), 50.5% female	100% Mexican-American	Daytime Sleepiness, Duration	Self-report daily sleep diaries	Parent-report, Neighborhood Criminal Events Scale	Neighborhood crime may interfere with healthful sleep durations
McKenzie et al., 2022	10,802	Ontario, CA	4–17 y M = 7.5 (2.27), sex not reported	60.6% White	Duration, Disturbance	Parent-report sleep problems, problems falling asleep, frequency of night waking, problems falling asleep after night waking, and child bed/wake time	Parent-report neighborhood antisocial behavior	Neighborhood antisocial behavior was associated with more problems falling asleep
Meldrum et al., 2018	7958	Florida, U.S.	M = 14.36 y, 53% female	37.75% White	Duration	Self-report sleep duration	Self-report feeling safe at school and in the neighborhood	Odds of insufficient sleep were 129% greater for adolescents who felt unsafe at school and in their neighborhood, 39% greater if they felt unsafe only at school, and 71% greater if they felt unsafe only in their neighborhood
Mousavi et al., 2024	133	Urban California, U.S.	12–16 y, M = 14.03 (1.35), 57.1% female	100% American Indian/Alaska Native	Efficiency, Duration, and Disturbance	School Sleep Habits Survey for Adolescents, Actigraphy	Self-reported neighborhood safety	Higher neighborhood safety was associated with less sleep disturbance and WASO, and higher sleep efficiency
Mrug et al., 2021	84	Birmingham, AL, U.S.	M = 13,16, and 17 y, 50% female	95% African American, 4% Caucasian, and 1% Hispanic	Disturbance	Adolescent Sleep Habit Survey	Self-report of exposure to violence	Community violence exposure at age 13 was predictive of increased sleep problems at age 16
Philbrook et al., 2020	219	Alabama, U.S.	M = 17.7 y (1.0), 55.3% female	69.9% White, 30.1% Black	Quality, Efficiency, and Duration	Actigraphy	Self-report community violence scale of the Community Experiences Questionnaire	Shorter sleep duration and poorer sleep quality were experienced more by African Americans who had less regulation of physiological arousal than their White counterparts
Piontak et al., 2017	151	California, U.S.	11–15 y M = 13 (0.91), 48.3% female	42.7% ethnic minority group (mostly Latinx)	Quality, Daytime Sleepiness, and Duration	Self-report number of hours slept, perceived sleep quality, and whether they “felt tired” (in replication participants reported sleep on sliding scale 1–100)	Self-report witnessed people fighting: at home, in school, in their neighborhoods, or “somewhere else” (in replication participants reported if they witnessed people arguing/physically fighting)	Tiredness in mornings after violence exposure despite no association between violence exposure and sleep duration or quality
Rubens et al., 2024	101	Large southeastern city, U.S.	16–24 y M = 17.86 (1.36), 53% female	100% Black or African American	Daytime Sleepiness, Efficiency, and Disturbance	Adolescent version of the Children’s Report of Sleep Patterns	Items from the adolescent version of the Childhood Trust Events Survey	No statistically significant findings
Spilsbury et al., 2014	46	Cleveland, OH, U.S.	8–16 y M=11.4 (2.4), 56.5% female	32.6% White, 60.9% African American	Timing, Efficiency, and Duration	Actigraphy, daily sleep diaries (child w/ parent help), parent-report Children’s Sleep and Health Questionnaire (CSHQ), and child report Self Sleep Report (SSR)	Self-report modified version of Recent Exposure to Violence Scale (REVS)	Witnessing homicide resulted in more sleep problems and WASO at baseline compared with a nonhomicide event. This was not sustained at 3-month follow-up.
Umlaut et al., 2015	263 (cross- sectional), 11,838 (longitudinal)	Mobile and Prichard, AL, U.S.	Cross-sectional analysis 14–15 y, longitudinal analysis 10–18 y, 49% female	100% African American	Daytime Sleepiness, Disturbance	Self-report bad dreams, trouble sleeping when bad things happen to a family member or friend, and Sleep-50 inventory	Self-report witnessing stabbing, shooting, or being cut	Exposure to violence impacts insomnia and sleep problems that are exacerbated by feelings of hopelessness
Umlaut et al., 2011	20,716	Mobile and Prichard, AL, U.S.	9.75–19.25 y, 36% female	99% African American	Daytime Sleepiness, Disturbance	Self-report 2 items from MYS Traumatic Stress Scale: bad dreams, trouble sleeping when bad things happen to a family member or friend	Self-report witnessing violence (cut, shot), victimization (brandishing weapon)	Sleep disturbance was affected for one year following witnessing a stabbing, shooting, or cutting
Wamser-Nanney & Chesher, 2018	276	Not stated	6–18 y M = 10.88 (3.39), 63.4% female	62.7% African American, 31.2% White, 5.1% Biracial, and 1.1% Hispanic	Daytime Sleepiness, Disturbance	Self- and caregiver report, select items related to sleep: 6 items from CBCL, 2 items from Trauma Symptom Checklist for young children, 2 items from MYS traumatic stress scale, 1 item from Trauma Symptom Checklist for children, and 2 items from UCLA PTSD index for DSM- IV child version	UCLA PTSD index for DSM-IV child version	Exposure to community violence was associated with increases in certain sleep difficulties
Zeringue et al., 2023	323	Small towns and semirural communities in Southeastern United States	M = 17.4 y (0.86), 52% female	60% White/European American, 40% Black/African American	Daytime Sleepiness, Efficiency, Duration, and Disturbance	School Sleep Habits Survey (sleep/wake subscale, sleepiness subscale), Actigraphy	Subscale of Neighborhood Environment Scale, items adapted from the Community Experiences Questionnaires	Community violence and neighborhood safety concerns are associated with self-report and objective sleep. The direction of this relationship depends on income status

NA/AI, Native American/American Indian; WASO, wake after sleep onset; CBCL, Child Behavior Checklist

**Table 3:** Quality assessment of included studies according to Joanna Briggs Institute Critical Appraisal Checklist

Author, year	Q1	Q2	Q3	Q4	Q5	Q6	Q7	Q8	Quality score

Bagley, 2016	Y	Y	Y	Y	Y	Y	Y	Y	Good
Bailey, 2005	Y	Y	Y	Y	Y	Y	Y	Y	Good
Cai, 2024	Y	Y	Y	Y	Y	Y	Y	Y	Good
Hall Brown, 2016	Y	Y	Y	Y	Y	Y	Y	Y	Good
Heissel, 2018	Y	Y	Y	Y	Y	Y	Y	Y	Good
Kliewer, 2019	Y	Y	Y	Y	Y	Y	Y	Y	Good
Langevin, 2024	Y	Y	Y	Y	Y	Y	Y	Y	Good
Lepore, 2013	Y	Y	Y	Y	Y	Y	Y	Y	Good
Lin, 2022	Y	Y	Y	Y	Y	Y	Y	Y	Good
MacKinnon, 2021	Y	Y	Y	Y	Y	Y	Y	Y	Good
McHale, 2011	Y	Y	Y	Y	Y	Y	Y	Y	Good
McKenzie, 2022	Y	Y	Y	Y	Y	Y	Y	Y	Good
Mousavi, 2024	Y	Y	Y	Y	Y	Y	Y	Y	Good
Mrug, 2021	Y	Y	Y	Y	Y	Y	Y	Y	Good
Philbrook, 2020	Y	Y	Y	Y	Y	Y	Y	Y	Good
Spilsbury, 2014	Y	Y	Y	Y	Y	Y	Y	Y	Good
Umlauf, 2015	Y	Y	Y	Y	Y	Y	Y	Y	Good
Zeringue, 2023	Y	Y	Y	Y	Y	Y	Y	Y	Good
Desch, 2023	Y	N	Y	Y	Y	Y	Y	Y	Fair
Jackson, 2024	Y	Y	Y	Y	Y	Y	N	Y	Fair
Kliewer, 2015	Y	Y	Y	N	Y	Y	N	Y	Fair
Piontak, 2017	Y	Y	N	Y	Y	Y	Y	Y	Fair
Rubens, 2024	Y	Y	N	Y	Y	Y	Y	Y	Fair
Umlauf, 2011	Y	Y	Y	Y	Y	Y	N	Y	Fair
Baiden, 2004	Y	Y	N	N	Y	Y	N	Y	Poor
Birkeland, 2022	Y	Y	N	N	Y	Y	N	Y	Poor
Dowdell, 2003	Y	Y	Y	Y	N	N	N	Y	Poor
Meldrum, 2018	Y	Y	N	N	Y	Y	N	Y	Poor
Wamser-Nanney, 2018	Y	N	N	N	Y	Y	N	Y	Poor

## Appendix A. Supporting information

Supplementary data associated with this article can be found in the online version at doi:10.1016/j.sleh.2025.10.009.
