# Supplementary material for: Associations between community violence and pediatric sleep health: A systematic review
Source: Sleep Health. Author manuscript; Available in PMC 2026 Jul 6. (PMC13334689; doi:10.1016/j.sleh.2025.10.009)
Supplement: Supplementary material [file NIHMS2178428-supplement-Supplementary_material.docx]

The following search was used across databases (CINAHL, PsycINFO, PubMed, SCOPUS): "Child, Preschool"[Mesh]) OR "Child"[Mesh] OR “Adolescent”[Mesh] OR “infant”[Mesh] OR child* OR preschooler OR toddler OR “school age” OR adolescen* OR teen* OR infant* OR baby OR pediatric* AND "Sleep"[Mesh] OR “Sleep Initiation and Maintenance Disorders”[Mesh] OR “circadian rhythm”[Mesh] OR “sleep wake disorders”[Mesh] OR “dreams”[Mesh] OR “sleep latency”[Mesh] OR “sleep quality”[Mesh] OR sleep* OR “circadian rhythm” OR “sleep stages” OR insomnia OR “sleep disturbance” OR “sleep health” OR awakeness OR nightmare* OR “sleep latency” OR “wake after sleep onset” OR “sleep quality” OR “sleep duration” AND "Violence"[Mesh] OR "Gun Violence"[Mesh] OR "Exposure to Violence"[Mesh] OR “crime”[Mesh] OR gun* OR firearm* OR shooting* OR robbery OR "gun violence" OR "gang*" OR "school violence" OR "neighborhood safety" OR "community safety" OR "neighborhood violence" OR "community violence" OR "police brutality" OR “police violence” OR “violent neighborhood” OR “exposure to violence” OR crime* OR “crime victim”.
